# Harnessing albumin as a carrier for the delivery of anti-HIV drugs to the lymphatic system

**DOI:** 10.1016/j.actbio.2025.11.017

**Published:** 2025-11-13

**Authors:** Ziqian Zhang, Yixian Huang, Raymond E. West, Deepika Mahesh, Patrick Joseph Oberly, Shichen Li, Zhangyi Luo, Yuang Chen, Haozhe Huang, Daniel J. Bain, Thomas D Nolin, Moses T. Bility, Robbie B Mailliard, Song Li

**Affiliations:** a Center for Pharmacogenetics, Department of Pharmaceutical Science, University of Pittsburgh School of Pharmacy, Pittsburgh, PA, USA; b UPMC Hillman Cancer Center, University of Pittsburgh, Pittsburgh, PA, USA; c Small Molecule Biomarker Core, University of Pittsburgh School of Pharmacy, Pittsburgh, PA, USA; d Department of Medicine, University of Pittsburgh School of Medicine, Pittsburgh, PA, USA; e Department of Geology and Environmental Science, University of Pittsburgh, Pittsburgh, PA, USA; f Department of Microbiology, Howard University College of Medicine, WA, DC, USA

**Keywords:** Antiretroviral delivery, Evans blue, Albumin, Lymph node, Nanomedicine

## Abstract

The persistence of human immunodeficiency virus (HIV) reservoirs in the lymphatic system remains a major obstacle to a complete cure, largely due to subtherapeutic concentrations of antiretrovirals (ARVs) in lymphatic tissues under current regimens. Developing innovative strategies to enhance ARV delivery to lymphatic tissues could therefore represent a paradigm shift in the pursuit of an HIV cure. Here, we describe a delivery strategy involving an Evans Blue (EB)-based prodrug conjugated with dolutegravir (DTG), a model ARV. This construct is designed to bind albumin with high affinity, facilitating transport to lymph nodes (LNs) via albumin-mediated trans-endothelial transcytosis. EB modification substantially improved the distribution of the conjugated molecule to LNs throughout the body, and the hEB-DTG prodrug increased DTG concentrations in LNs by up to 10-fold, suggesting a promising strategy for eradicating HIV reservoirs.

## 1. Introduction

The human immunodeficiency virus (HIV) pandemic, now in its fifth decade since the first reported cases in 1981, continues to affect approximately 40 million people globally [1–3]. While numerous antiretrovirals (ARVs) targeting key stages of the HIV life cycle have been approved and form the basis of combination antiretroviral therapy (cART) [3–6], a cure remains elusive [6,7]. cART has transformed HIV into a manageable chronic condition, allowing people with HIV to achieve a near-normal life expectancy with consistent adherence [4,7–9]. Nevertheless, despite reducing plasma viral loads to undetectable levels, cART does not eradicate the virus. Latent HIV reservoirs persist in lymph nodes (LNs) and other tissues [10–12], and discontinuation of therapy or the development of ARV resistance leads to rapid viral rebound from these reservoirs. The lymphatic system, particularly gut-associated lymphoid tissue (GALT) and LNs, harbors the highest latent viral loads and is a major source of viral rebound [12–14]. A leading hypothesis posits that the establishment and maintenance of these reservoirs are due to insufficient ARV concentrations in the lymphatic system, as evidenced by persistent viral replication in these sanctuaries [15–17]. Consequently, the lymphatic system is a critical target for eradication strategies, creating an urgent need for innovative delivery systems that can enhance ARV concentrations within these tissues.

The suboptimal drug concentrations observed in the lymphatic system are largely attributable to its unique anatomy and physiology. For small-molecule drugs in the systemic circulation, access to lymphatic tissues involves a multi-step process: extravasation from blood capillaries into the interstitial space, entry into initial lymphatic capillaries, and subsequent transport to LNs [18]. While ARVs readily diffuse into the interstitial space, their entry into lymphatic capillaries is limited, as lymphatic drainage occurs at a much slower rate than venous reabsorption [19]. In addition, the architecture of the lymphatic system favors the retention of larger molecules; particles larger than 10 nm or with a molecular weight exceeding 16 kDa are effectively retained due to the low permeability of blood capillaries to such macromolecules [20,21]. Furthermore, small molecules that do reach the LNs are often rapidly cleared back into the bloodstream via efferent lymphatic flow. Collectively, these factors contribute to persistently low drug concentrations in LNs, thereby limiting the efficacy of ARVs against localized HIV reservoirs.

To overcome this pharmacological barrier, nanoparticle (NP)-based delivery systems have been explored. These systems typically encapsulate ARVs for subcutaneous (*s.c.*) injection, facilitating direct lymphatic transport and promoting drug accumulation in LNs with sustained release profiles [22–26]. A key limitation of this approach, however, is that the majority of administered NPs accumulate primarily in the local draining LNs near the injection site. Although some studies have demonstrated that certain NPs can achieve intralymphatic retention and broader systemic distribution [27–29], drug concentrations in distant LNs remain substantially lower. This uneven distribution hinders the eradication of latent viruses in remote lymphoid tissues. Consequently, a critical unmet need exists for delivery platforms capable of uniformly elevating ARV concentrations across the entire lymphatic system to effectively target more HIV reservoirs in LNs and GALTs.

In this study, we investigated Evans Blue (EB) conjugates of dolutegravir (DTG) as a strategy to enhance LN delivery. This approach leverages the high binding affinity of EB for albumin, an endogenous carrier protein known to extravasate into tissues via transcytosis across endothelial barriers [30,31]. We hypothesized that following intravenous (*i.v.*) administration, EB-DTG conjugates would rapidly bind circulating albumin, thereby hijacking this natural transport pathway to facilitate efficient DTG delivery to LNs. To evaluate this hypothesis, we quantified drug concentrations in LNs using high-performance liquid chromatography-mass spectrometry (HPLC-MS) and assessed the antiviral efficacy of the EB conjugate.

## 2. Materials and methods

### 2.1. Materials

1-amino-naphthol-2,4-disulfonic acid monosodium salt, succinic acid, succinic anhydride, cisplatin, were purchased from Tokyo Chemical Industry CO., LTD. (OR, USA). Chloroacetyl chloride were purchased from Thermo Fisher Scientific (MA, USA). Dolutegravir was purchased from Advanced ChemBlocks Inc. (CA, USA). Filipin, Dulbecco’s Modified Eagle’s Medium (DMEM), o-Tolidine, Evans Blue and C_18_-reversed phase silica gel were purchased from Sigma-Aldrich (MO, USA). Vascular cell basal medium, endothelial cell growth supplement, heat-inactivated fetal bovine serum (FBS), penicillin-streptomycin solution, and all antibodies for flow cytometry were purchased from Invitrogen (NY, USA). All solvents used in this study were of HPLC grade.

### 2.2. Cell lines

HEK293T and HUVEC cell lines used in this work were obtained from ATCC (VA, USA). TZM-bl cells (also called JC53BL-13) were obtained from the NIH AIDS Research and Reference Reagent Program (Cat. no. 8129). HEK293T were cultured in DMEM medium supplemented with 10 % FBS and penicillin/streptomycin (100 U·mL^−1^). HUVEC primary umbilical vein endothelial cells were maintained in vascular cell basal medium supplemented with endothelial cell growth supplement, 10 % FBS and penicillin/streptomycin (100 U·mL^−1^). TZM-bl cells were cultured in DMEM with L-glutamine, sodium pyruvate, glucose, pyridoxine and 25 mM HEPES (4-(2-hydroxyethyl)-1-piperazineethanesulfonic acid) containing 10 % FBS and penicillin/streptomycin (100 U·mL^−1^). All cells were cultured at 37 °C in a humidified atmosphere with 5 % CO_2_.

### 2.3. Animals

Female BALB/c mice (4–6 weeks) were purchased from The Jackson Laboratory (ME, USA). All animals were housed under pathogen free conditions according to the Association for Assessment and Accreditation of Laboratory Animal Care (AAALAC) guidelines. All animal-related experiments were performed in full compliance with institutional guidelines and approved by the Animal Use and Care Administrative Advisory Committee at the University of Pittsburgh. Mice were housed at an ambient temperature of 22 °C (22–24 °C) and humidity of 45 %, with a 14/10 day/night cycle (on at 6:00, off at 20:00), and allowed access to food *ad libitum*.

### 2.4. Synthesis of hEB and hEB-platin

The synthesis of half-truncated Evans Blue (hEB) was reported elsewhere [32].

As for hEB-platin, cisplatin was first oxidated by H_2_O_2_ to get oxidated-cisplatin as reported [33]. Then 1.320 g succinic anhydride was added to a suspension of 1.075 g oxidated-cisplatin in 20 mL DMF, and the reaction mixture was stirred at 70 °C for 1.5 h. During this time, the solid material dissolved to form a yellow solution. DMF was then removed under reduced pressure. The residue was dissolved in acetone and filtered to give a clear, yellow solution. This solution was concentrated under reduced pressure, and the subsequent addition of diethyl ether led to the precipitation of a pale-yellow solid. The product was dried in vacuum to obtain cisplatin-2SA.

One hundred and forty (140) mg cisplatin-2SA, 77 mg N–Hydroxy-succinimide, 126 mg 1-ethyl-3-(3-dimethylaminopropyl) carbodiimide, 150 μL triethylamine were added to 15 mL dimethylformamide and stirred for 1 h. Then 145 mg hEB was added and reacted under 37 °C for 48 h. Then the mixture was purified through dialysis in water and lyophilization to obtain hEB-platin (89 mg, 32 %).

#### hEB-platin:

^1^H NMR (400 MHz, D_2_O) δ 8.25 (s, 1H), 7.63 – 7.57 (m, 1H), 7.56 (d, J = 2.2 Hz, 1H), 7.53 – 7.48 (m, 1H), 7.47 – 7.42 (m, 1H), 7.34 (s, 1H), 7.28 (t, J = 9.5 Hz, 2H), 7.15 (d, J = 9.9 Hz, 1H), 6.98 (d, J = 8.2 Hz, 1H), 2.71 – 2.59 (m, 4H), 2.51 (q, J = 7.0 Hz, 4H), 2.34 (s, 2H), 2.21 (s, 2H), 2.06 (s, 2H). Pt content tested by ICP-MS: 18.1 % (w/w).

### 2.5. Synthesis of hEB-DTG

To the solution of 50 mg DTG in anhydrous 10 mL dimethylformamide was added 60 mg N,N-Diisopropylethylamine. The reaction mixture was stirred at 0 °C for 30 min. 27 mg chloro-acetyl chloride was added to the reaction mixture and stirred at 0 °C for 10 min and slowly raised to room temperature. The reaction mixture was stirred for 16 h at room temperature then water was added to quench the reaction. The mixture was extracted with dichloromethane. The organic phase was separated and washed with saturated sodium bicarbonate, water and brine, and dried over anhydrous sodium sulfate. Filtration and removal of the solvent under reduced pressure provided the crude compound, which was purified by silica gel chromatography to obtain the product DTG-Cl (46 mg, 78 %). 50 mg DTG-Cl, 25 mg hEB and 70 mg potassium carbonate were added to 10 mL ethanol and stirred at 40 °C for 16 h. Then the hEB-DTG (21 mg, 45 %) was obtained after purifying the mixture by C_18_ reverse silica gel chromatography and removing solvent by lyophilization.

#### DTG-Cl:

^1^H NMR (400 MHz, CDCl_3_): δ 10.16 (t, J = 6.0 Hz, 1H), 8.62 (s, 1H), 7.40 – 7.23 (m, 1H), 6.81 (q, J = 9.0, 8.1 Hz, 2H), 5.22 (t, J = 4.8 Hz, 1H), 4.89 (p, J = 6.8 Hz, 1H), 4.60 (d, J = 5.9 Hz, 2H), 4.50 (s, 2H), 4.44 (dd, J = 13.6, 3.8 Hz, 1H), 4.25 (dd, J = 13.8, 5.8 Hz, 1H), 4.11 (q, J = 7.2 Hz, 1H), 3.95 (d, J = 8.4 Hz, 2H), 2.16 (dt, J = 15.2, 5.8 Hz, 1H), 1.51 (d, J = 14.1 Hz, 1H), 1.32 (d, J = 7.1 Hz, 3H), 1.25 (t, J = 7.1 Hz, 1H). MS: Calculated for [M + H]^+^ C_22_H_20_ClF_2_N_3_O_6_, 496.1087; found 496.1093.

#### hEB-DTG:

^1^H NMR (400 MHz, MeOD): δ 8.45 (s, 2H), 7.83 – 7.76 (m, 2H), 7.67 (t, J = 7.5 Hz, 1H), 7.60 (d, J = 7.4 Hz, 1H), 7.53 (d, J = 34.4 Hz, 1H), 7.35 (s, 2H), 7.29 (d, J = 10.1 Hz, 1H), 7.23 (s, 1H), 7.20 (d, J = 7.9 Hz, 1H), 6.81 (d, J = 10.8 Hz, 1H), 6.69 (d, J = 8.3 Hz, 1H), 5.11 (s, 1H), 4.48 (s, 1H), 4.18 (d, J = 14.4 Hz, 1H), 4.00 (d, J = 16.1 Hz, 1H), 3.84 (d, J = 2.1 Hz, 1H), 3.81 – 3.73 (m, 1H), 2.52 (s, 3H), 2.14 (s, 3H), 1.94 (s, 1H), 1.36 (d, J = 13.6 Hz, 1H), 1.21 (d, J = 7.2 Hz, 3H).

### 2.6. Characterization of the synthesized compounds

^1^H NMR spectrums were examined on a Bruker Ascend 400 NMR system at 400.0 MHz with CDCl_3_, MeOD, D_2_O or N,N-Dimethylformamide-d7 as the solvent.

Fourier transform infrared spectroscopy spectrums were examined on a Thermo Nicolet iS50 FTIR with PM-IRRAS and VCD.

Mass spectrums were examined on a Bruker Daltonics UltrafleXtreme MALDI TOF-TOF.

### 2.7. Gel retardation study

Twenty (20) μg EB, hEB, hEB-platin, or hEB-DTG were mixed with or without various amounts of BSA, globulin, fibrinogen or FBS in 200 μL saline water, then 40 μL of the mixed solution were loaded to 1.5 % agarose gel and run under 120 V for 30 min.

### 2.8. Cellular uptake study in vitro

HEK293T or HUVEC cells were seeded to 6-well plates. After overnight incubation, the culture medium was replaced by fresh medium containing 10 μM EB and 2, 5, or 20 mg/mL BSA for incubation of 0.5, 1 or 3 h at 37 °C. Later, cells were washed with cold PBS and fixed with PBS containing 4 % (w/v) formaldehyde.

Cellular uptake of different treatments was quantified by flow cytometry. Fluorescence was examined at an excitation wavelength of 532 nm and an emission wavelength of 660 nm. 10 × 10^4^ events were collected for each sample.

Cells were stained with FITC- early endosome antigen 1 (EEA1) antibody and DAPI for fluorescent microscopy.

### 2.9. Transwell study

HEK293T were seeded to 6-well plates as lower chamber and HUVEC were seeded to Corning (NY, USA) 6-well plate inserts (0.4 μm pore size) in another plate as upper chamber, respectively. After incubation and the cell layer on the upper chamber became intact, the culture medium in the inserts was replaced by fresh medium containing 0, 5 or 20 μg/mL filipin and 0 or 10 μg/mL LPS for overnight incubation. Next, the culture medium in the inserts was replaced with fresh medium containing 10 μM EB, hEB or hEB-DTG and 2 mg/mL BSA, then the inserts were moved to the wells cultured with HEK293T cells for incubation of 8 h at 37 °C. After that, HEK293T cells in the lower chambers were washed with cold PBS.

EB signals in HEK293T cells were quantified by flow cytometry. Fluorescence was examined at an excitation wavelength of 532 nm and an emission wavelength of 660 nm. 10 × 10^4^ events were collected for each sample.

### 2.10. Distribution of EB, hEB and hEB-DTG in LN tissue and cells in LNs

Five (5) μM/kg EB, hEB or hEB-DTG were *i.v.* injected into mice pretreated intraperitoneally with or without 2.5 mg/kg LPS for 24 h. After 24 h, LNs from different sites were collected and their signals of EB were checked under fluorescent microscopy. To evaluate the EB signals in various types of cells, LNs were disrupted mechanically using scissors, digested with a mixture of deoxyribonuclease I (0.3 mg/mL, Sigma-Aldrich) and TL Liberase (0.25 mg/mL, Roche, IN, USA) in serum-free RPMI-1640 at 37 °C for 30 min, and dispersed through a 40 μm cell strainer (BD Biosciences, CA, USA). After red blood cell lysis, live/dead cell discrimination was performed using a Zombie NIR Fixable Viability Kit (BioLegend, CA, USA, dilution: 1/1000) at 4 °C for 30 min in DPBS. Surface staining was performed at 4 °C for 30 min in FACS staining buffer (1 × phosphate-buffered saline/5 % FBS/0.5 % sodium azide) containing designated antibody cocktails (PerCP anti-mouse CD45 antibody, Brilliant Violet 785 anti-mouse CD4 antibody, FITC anti-mouse CD8 antibody, APC anti-mouse CD11b antibody, APC/Cyanine7 anti-mouse F4/80 antibody, Pacific Blue anti-mouse CD19 antibody and AF647 anti-mouse CD27 antibody; dilution: 1/200 for all antibodies). Flow cytometry was performed with LSRII (BD Biosciences) and Aurora (Cytek Biosciences, CA, USA) instruments and analyzed by FlowJo (BD Biosciences). The EB signals in each type of cell were quantified as mentioned above.

### 2.11. Quantification of Pt by ICP-MS in LNs

Five (5) mg/kg cisplatin or hEB-platin, lipoplatin containing equivalent amount of Pt were *i.v.* or *s.c.* injected into mice. After 24 h, organs and LNs from different sites were collected. Samples were placed into a pre-weighed Purillex PFA bottle (Savillex, MN, USA) and the net weights were recorded. Plasma and tissue samples were freeze at −80 °C and lyophilized. 4 mL HNO_3_ (69.0 % w/w) and 2 mL HCl (37 % w/w) were added into each PFA bottle, which was then immersed into 90 °C water bath for sample digestion to obtain free Pt ion in the lysates. The lysates were then dried down for 12 h at 50 °C to get rid of residual acid. HCl (5 %) was added, and the samples were transferred into a 15 mL centrifuge tube and subsequently Pt concentrations in the samples were measured using a PerkinElmer Nexion 300x Inductively Coupled Plasma-Mass Spectrometer. A standard curve for Pt on the ICP-MS was created by diluting a Pt single element standard (1000 μg/mL, for AA and ICP, Spex CertiPrep, NJ, USA).

### 2.12. Size distribution of albumin and hEB-DTG bound albumin

The size distribution of albumin and hEB-DTG bound albumin were measured via dynamic light scattering by a Malvern Zetasizer Nano ZS (Malvern Instruments Ltd, Malvern, U.K.).

### 2.13 . PK study and quantification of DTG by HPLC-MS in serum and LNs

Five (5) mg/kg DTG, or hEB-DTG containing equivalent amount of DTG were *i.v.* injected into mice. After 1, 4, 12, 24 or 48 h, serum and LNs were collected. Samples were homogenized and vortex in 1 mL mixture of water and methanol (1/1, v/v), and then another 1 mL mixture of methanol and acetonitrile (1/1, v/v) was added for extraction. After centrifugation at 12,500 g for 10 min, the supernatants were collected for measurement under HPLC-MS with a previously reported method [34].

For hydrolysis of the ester bond in hEB-DTG to release free DTG, the biological samples suspension mentioned above were incubated in 300 mg/mL guanidine hydrochloride solution at 80 °C for 24 h before extraction.

### 2.14. Cell viability assay

DTG and hEB-DTG prodrug cytotoxicity was measured at different concentrations against TZM-bl reporter cell line in the presence or absence of HIV-WT virus using the CellTiter-Glo^™^ 2.0 Assay (Promega, WI, USA) according to the manufacturer’s protocol. TZM-bl cells were seeded in the 96-well white plate at a density of 1 × 10^4^ cells/well, and after 24 h, the cells were infected with HIV-WT virus and treated with DTG and hEB-DTG in a concentration range (diluted 1:100) from 3 × 10^−1^ mg/mL to 3 × 10^−15^ mg/mL. After 48 h, cells were subjected to CellTiter-Glo^™^ 2.0 Assay (Promega). The CellTiter-Glo^™^ 2.0 Reagent was equilibrated to room temperature and gently mixed. 100 μL of CellTiter-Glo^™^ 2.0 Reagent was added to each well containing 100 μL of cells in the medium. Contents were shaken in an orbital shaker for 2 min and incubated at room temperature for precisely 10 min, and then luminescence was measured with a Varioskan LUX Multimode Microplate Reader. Untreated/infected cells were used as a baseline control. Viability of cells was presented as a percentage of OD values of the control cells. For each assay run, data points were collected in triplicate.

### 2.15. Drug susceptibility assay

DTG and hEB-DTG luciferase expression levels were measured at different concentrations against TZM-bl reporter cell line in the presence of HIV-WT virus using the Britelite Plus Ultra-High Sensitivity Luminescence Reporter Gene Assay System (Revvity, MA, USA) according to the manufacturer’s protocol. TZM-bl cells were seeded in clear bottom white 96-well plate in a total volume of 200 μl DMEM-10 complete (+ 10 % FBS + 1 % penicillin/streptomycin) at a density of 1 × 10^4^ cells/well. After 24 h, the cells were infected with the HIV-WT virus and treated with DTG and hEB-DTG in a concentration range (serial diluted 1:100) from 3 × 10^−1^ mg/mL to 3 × 10^−15^ mg/mL in PRF-10 complete media (+10 % FBS + 1 % penicillin/streptomycin). After 48 h of incubation, the cells were subjected to Britelite Plus Ultra-High Sensitivity Luminescence Reporter Gene Assay System (Revvity). The Britelite plus Lyophilized Substrate was reconstituted with the given Britelite Plus Reconstitution Buffer. After gently inverting and settling the solution for 5 min, 100 μL of the reconstituted Britelite Substrate solution was added to each well containing 100 μL of cells in the medium. Luminescence was measured with a Varioskan LUX Multimode Microplate Reader precisely after 2 min. Untreated/infected cells were used as baseline control. The inhibitory concentrations of each drug were presented as a percentage of OD value of the control cells. For each assay run, data points were collected in triplicate.

### 2.16. Statistical analysis

Statistical analysis was performed with two-tailed Student’s *t*-test for comparison between two groups and one-way analysis of variance (ANOVA) for comparison among multiple groups. Results were considered statistically significant if P < 0.05. Prism 9.4.0 (GraphPad Software) was used for data analysis and graph plotting.

## 3. Results and discussion

### 3.1. EB strongly binds to albumin for cellular uptake, transcytosis and LN distribution

EB, a dye known for its strong binding affinity to albumin [35,36], was selected as a ligand for developing ARV prodrugs. Following administration, EB rapidly associates with circulating albumin, with each albumin molecule capable of binding up to 14 EB molecules, ensuring that the majority is in a bound state in the bloodstream [37]. This EB-albumin complex is subsequently distributed throughout the body, a property historically exploited for assessing blood-brain barrier permeability and for targeted delivery to albumin-rich tissues [36,37]. Chen’s group has reported EB-based tumor imaging and therapeutic agents by capitalizing on albumin’s tumor-homing effect [30,31,37]. However, the application of EB-based prodrugs for targeted delivery to lymphatic tissues remains largely unexplored.

**Fig. 1.**
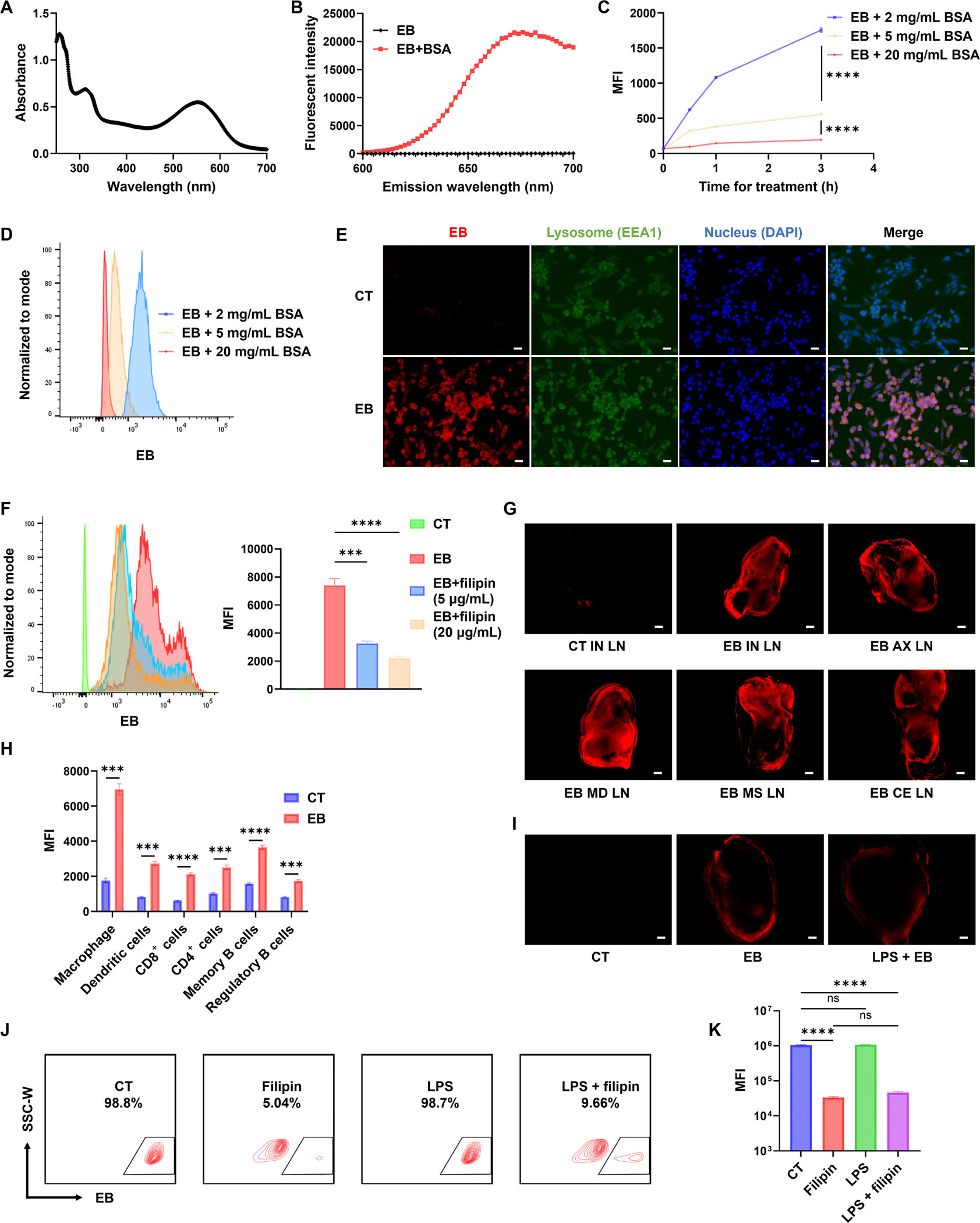
EB strongly binds to albumin for cellular uptake, transcytosis and LN distribution. (A) UV–Vis absorbance spectrum of EB from 250 to 700 nm. (B) Fluorescence emission spectrum of EB (excitation: 560 nm) in the presence or absence of BSA (10 times molar ratio). (C&D) Flow cytometric analysis of EB uptake in HEK293T cells incubated with EB and increasing concentrations of BSA. Shown are the time-dependent changes in EB signal (C), and the representative histograms from cells treated for 3 h (D). N = 3. MFI: mean fluorescence intensity. (E) Fluorescence micrograph of HUVECs showing intracellular EB. Scale bar: 20 μm. (F) Evaluation of EB transport across a HUVEC monolayer in a transwell assay, with or without pretreatment with the transcytosis inhibitor filipin. N = 3. (G) Fluorescence images of LNs harvested from various anatomical sites 24 h post *i.v.* EB administration. Scale bar: 200 μm. (H) Flow cytometric analysis of EB uptake by different immune cell populations isolated from LNs. N = 3. (I) Fluorescence images of LNs from naive and LPS-treated mice 24 h post *i.v.* EB injection. Scale bar: 200 μm. (J&K) Effect of filipin and LPS on EB transport across a HUVEC monolayer. Shown are the representative flow cytometry plots of cells in the lower chamber (J) and the MFI in each group (K). N = 3. Data are presented as mean ± SEM. Statistical analysis was performed by two-tailed Student’s t-test for comparison between two groups and one-way ANOVA for comparison among multiple groups, ns, no significance, *P < 0.05, **P < 0.01, ***P < 0.001, ****P < 0.0001.

We first evaluated the LN-homing propensity of EB following its binding to bovine serum albumin (BSA). The UV–visible absorbance spectrum of free EB exhibited a distinct peak at approximately 560 nm (Fig. 1A). Excitation at this wavelength, after mixing EB with BSA, yielded a strong fluorescence emission (Fig. 1B). The pronounced increase in emission intensity upon binding confirms a strong interaction between EB and BSA. This characteristic excitation and emission profile provides a convenient method for tracking EB molecules.

A gel retardation assay confirmed that the majority of EB molecules were bound to albumin at albumin/EB weight ratios of 25 or higher, demonstrating the high loading capacity of albumin for EB transport (Figure S1). This interaction was specific to albumin, as a parallel assay showed no detectable binding of EB to globulin or fibrinogen, two other major serum proteins (Figure S2). These findings indicate that in albumin-rich environments like blood and cell culture medium, EB exists predominantly in its albumin-bound form. Furthermore, the stable and intense fluorescence of the albumin-bound EB complex provides a reliable method for quantifying EB concentration.

Cellular uptake of EB was found to be both efficient and albumin-dependent. This was demonstrated by a significant reduction in intracellular EB levels upon the addition of excess albumin to the culture medium (Fig. 1C and D), suggesting that the availability of free albumin in the environment competes with the cellular uptake process. Fluorescence microscopy further revealed a substantial colocalization of the EB signal with a lysosomal marker, indicating that internalization occurs primarily via endocytosis (Fig. 1E).

We next investigated the ability of EB to traverse an endothelial barrier using a transwell system. In this model, a confluent monolayer of HUVECs, which mimics the vascular endothelium, separated the upper and lower chambers. This setup prevents the passive intercellular diffusion of large molecules like albumin. When albumin-bound EB was applied to the upper chamber, strong fluorescent signals were detected in the lower chamber after 8 h, indicating successful transport. Importantly, this transport was significantly and dose-dependently inhibited when the HUVECs were pretreated with filipin, a known inhibitor of transcytosis. These results demonstrate that EB-albumin complexes cross the endothelial layer primarily via transcytosis (Fig. 1F).

Finally, to evaluate the *in vivo* distribution profile, EB was administered *i.v.* to mice, and LNs from various anatomical sites were harvested 24 h post-injection for fluorescence imaging. Robust EB fluorescence was detected in all LNs examined, demonstrating its widespread distribution throughout the lymphatic system (Fig. 1G). Flow cytometry analysis of cells isolated from these LNs confirmed significant EB signal across multiple cell populations, verifying the effective uptake of the albumin-bound complex by the resident cells in LNs (Figs. 1H and S3).

Given that systemic inflammation during viral infection can enhance macromolecular extravasation, we evaluated whether inflammatory conditions alter EB distribution to LNs. We induced systemic inflammation using lipopolysaccharide (LPS) from *Escherichia coli* to mimic the acute phase of HIV infection. *In vivo*, EB signals in LNs from LPS-treated mice were not significantly different from those in naive mice 24 h post-injection (Fig. 1I). We further investigated the effect of inflammation on EB transport using our *in vitro* transwell model. HUVEC monolayers were pretreated with filipin, LPS, or both before adding EB. Flow cytometric analysis of cells in the lower chamber showed that LPS treatment alone did not enhance EB transport across the endothelium (Figs. 1J and K). Furthermore, filipin inhibited EB transport to a similar extent in both naive and LPS-treated HUVECs, confirming that transcytosis remains the dominant mechanism for EB-albumin transport, irrespective of inflammatory status.

### 3.2. EB conjugated small molecules display dramatic improvements in LN distribution

**Fig. 2.**
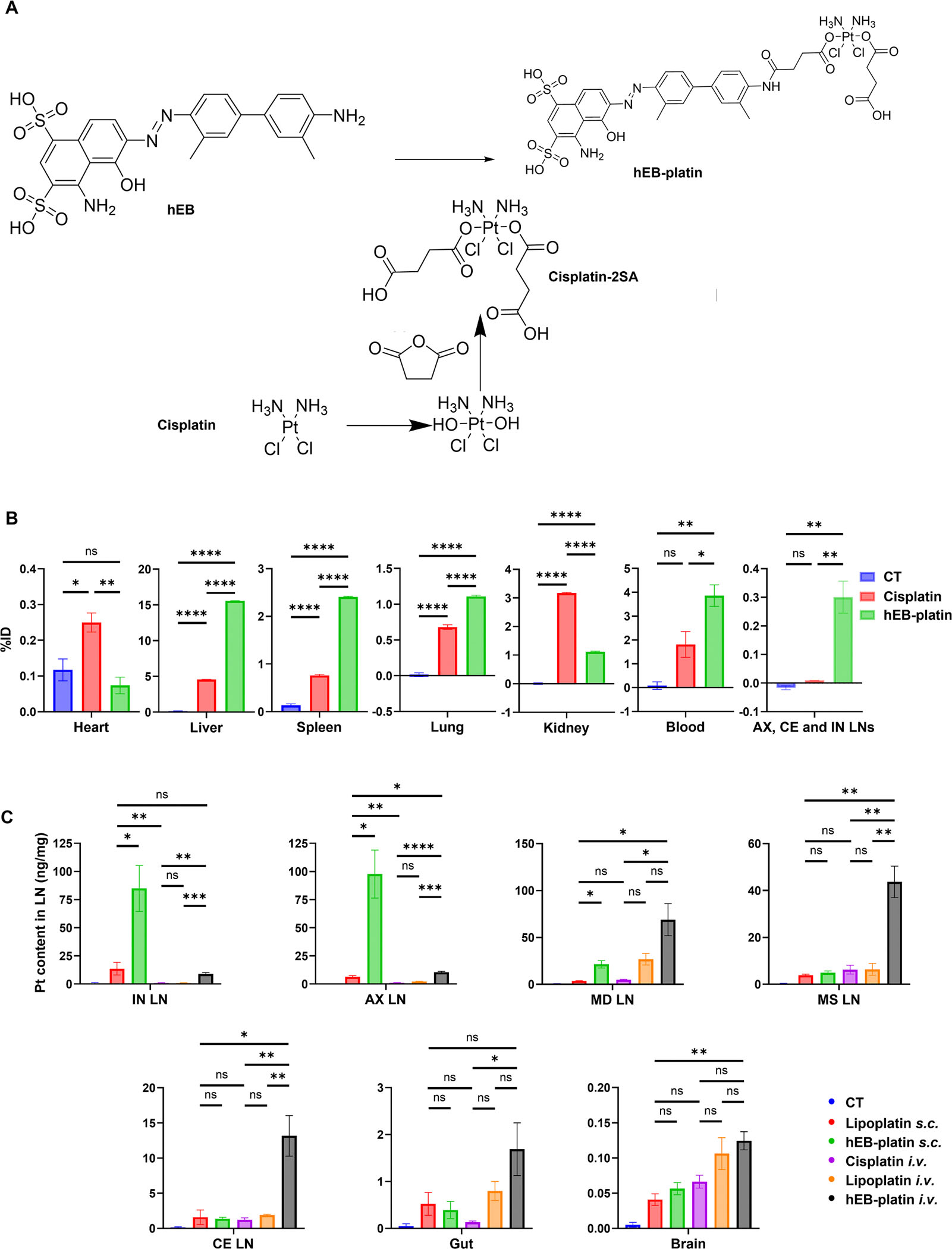
EB conjugated small molecules showed dramatic improvements in LN distribution. (A) Synthesis scheme of hEB-platin. (B) Biodistribution of Pt in select organs and pooled LNs 24 h post-*i.v.* injection of free cisplatin or hEB-platin, quantified by ICP-MS. N = 3. (C) Comparative Pt distribution across individually collected LNs and tissues 24 h post-injection of free cisplatin, lipoplatin (*s.c.*), lipoplatin (*i.v.*), hEB-platin (*s.c.*), or hEB-platin (*i.v.*), quantified by ICP-MS. N = 3. Data are presented as mean ± SEM. Statistical analysis was performed by one-way ANOVA for comparison among multiple groups, ns, no significance, *P < 0.05, **P < 0.01, ***P < 0.001, ****P < 0.0001.

We next investigated whether conjugating a model drug to EB could enhance its lymphatic delivery. We selected cisplatin for this purpose, as its platinum (Pt) content can be quantified directly in tissues using inductively coupled plasma-mass spectrometry (ICP-MS), eliminating the need for complex extraction procedures. Cisplatin was first oxidized and reacted with succinic anhydride to generate cisplatin-2SA (Fig. 2A), which was then conjugated to a half-truncated EB derivative (hEB) to form hEB-platin. The structure of hEB-platin was confirmed by nuclear magnetic resonance (NMR; Figure S4). A gel retardation assay confirmed that, like EB, both hEB and hEB-platin retained strong binding affinity for BSA (Figure S5).

To evaluate the impact of hEB conjugation on lymphatic targeting, mice were administered either free cisplatin or hEB-platin. Tissues were harvested 24 h post-injection, and Pt content was analyzed by ICP-MS. LNs from the axillary (AX), cervical (CE), and inguinal (IN) regions were pooled for analysis. As shown in Fig. 2B and S6, mice treated with hEB-platin exhibited reduced Pt contents in heart and kidneys but higher Pt levels in blood, liver, spleen, and lungs, indicating prolonged systemic circulation. Most notably, the Pt content in the pooled LNs was over 20-fold higher in the hEB-platin group compared to the free cisplatin group, demonstrating that hEB conjugation dramatically enhances the delivery of cisplatin to the lymphatic system.

We next sought to determine if systemic administration of hEB-based prodrug strategy could achieve broad delivery of a therapeutic to multiple, discrete LNs. Mice received *i.v.* injections of free cisplatin, cisplatin-loaded liposomes (lipoplatin), or hEB-platin. After 24 h, various tissues—including individually collected IN, AX, mediastinal (MD), mesenteric (MS), and CE LNs, as well as gut and brain—were analyzed for Pt content. As shown in Fig. 2C, hEB-platin administration resulted in Pt levels that were over 10-fold higher in all collected LNs compared to free cisplatin, confirming that the hEB prodrug enables widespread LN targeting via the vascular route. Interestingly, a substantial increase in Pt content was also observed in the gut, but not in the brain. For lipoplatin, it showed improved Pt concentrations in the MD LN and gut compared to free cisplatin, likely attributable to its enhanced retention in the body. While the Pt concentrations in these two tissues were not statistically different between the hEB-platin and lipoplatin groups, hEB-platin achieved significantly higher drug concentrations in all other LNs examined.

NPs have been reported to enhance the delivery of HIV drugs to LNs via *s.c.* injection [22–26]. We therefore included this route of administration for a direct comparison. Mice received *s.c.* injections of lipoplatin or hEB-platin in the abdomen, and drug concentrations in individual LNs were compared to those in mice receiving *i.v.* hEB-platin. As shown in Fig. 2C, *s.c.* injection of lipoplatin significantly elevated Pt content in local draining LNs (IN and AX LNs). However, Pt levels in distant LNs were comparable to those achieved with *i.v.* free cisplatin, indicating that *s.c.* administered NPs (~150 nm) did not effectively distribute drugs to the broader lymphatic system. This limited distribution could hinder the effectiveness in eradicating widely disseminated HIV reservoirs. Notably, even in the local draining LNs, the Pt content in lipoplatin group did not exceed that achieved by *i.v.* hEB-platin. Mice receiving *s.c.* hEB-platin showed the highest Pt accumulation in local draining LNs, significantly surpassing levels from *s.c.* lipoplatin. However, similar to lipoplatin, this *s.c.* strategy did not enhance delivery to distant LNs. Taken together, these data demonstrate that *i.v.* administration of hEB-based prodrugs provides an effective method for enhancing drug distribution across the entire lymphatic system. Given that latent HIV reservoirs are widely distributed throughout the body, our systemic delivery strategy offers an advantage over localized *s.c.* approaches for targeting and potentially eliminating these viral sanctuaries.

### 3.3. Development and characterization of hEB-DTG

**Fig. 3.**
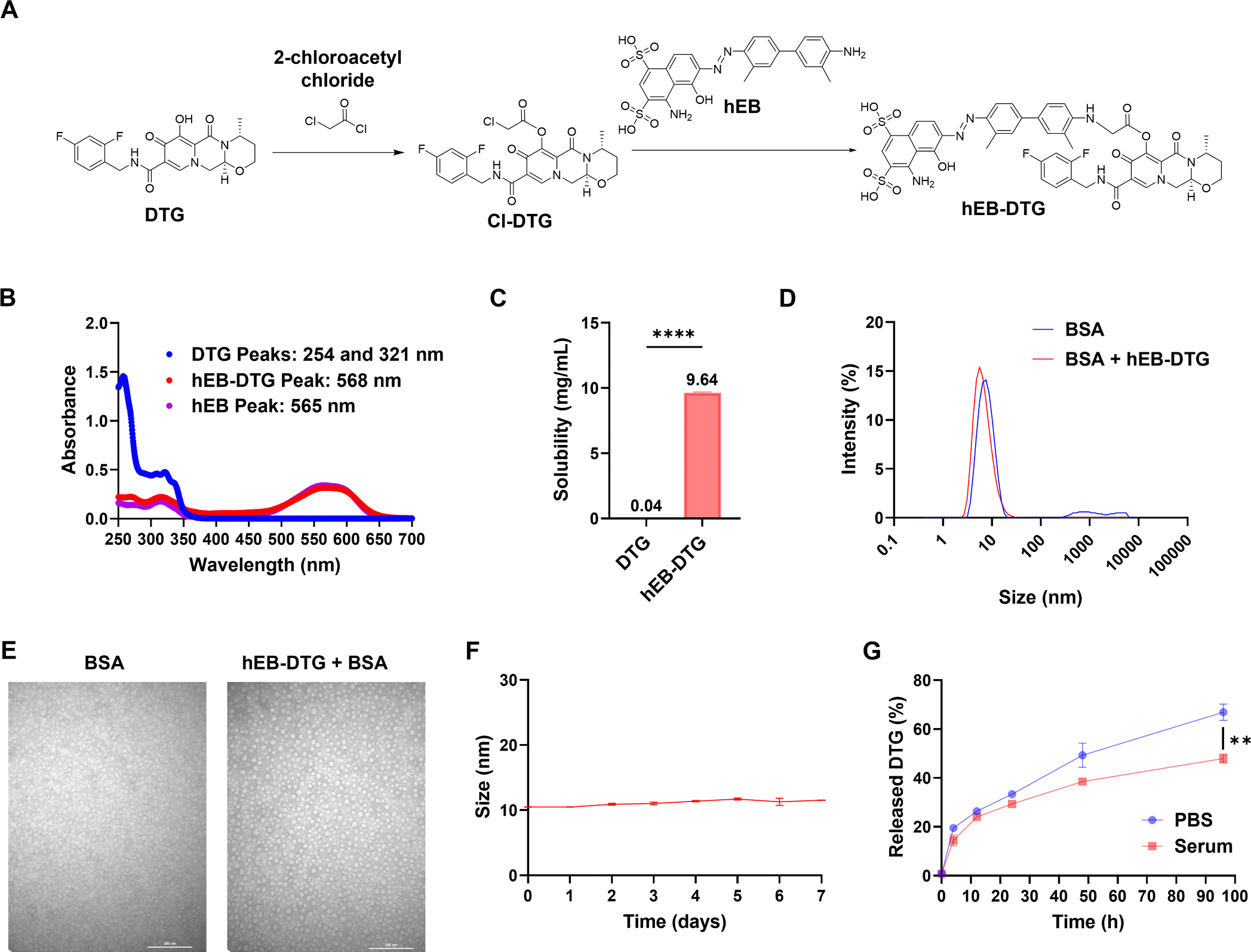
Synthesis and characterization of hEB-DTG. (A) The synthesis route of hEB-DTG. (B) UV–Vis absorption spectrum of DTG, hEB and hEB-DTG. (C) Aqueous solubility of DTG and hEB-DTG. N = 3. (D) Hydrodynamic size distribution (by intensity) of free BSA and hEB-DTG bound BSA, measured by dynamic light scattering. (E) Representative TEM images of BSA and hEB-DTG bound BSA. Scale bar: 200 nm. (F) Stability of the hEB-DTG bound BSA over 7 days at room temperature, presented as mean hydrodynamic diameter. N = 3. (G) *In vitro* release kinetics of DTG from the hEB-DTG prodrug incubated at 37 °C in PBS or serum, expressed as cumulative release percentage. N = 3. Data are presented as mean ± SEM. Statistical analysis were performed by two-tailed Student’s t-test for comparison between two groups, ns, no significance, *P < 0.05, **P < 0.01, ***P < 0.001, ****P < 0.0001.

Having established that hEB conjugation enhances lymphatic delivery of a model drug (cisplatin), we applied this strategy to DTG, an integrase inhibitor. The aromatic -OH group on DTG served as a potential site for hEB modification. 2-chloroacetyl chloride was successfully introduced between DTG and hEB, yielding the hEB-DTG prodrug (Fig. 3A). The structures of the synthesized compounds were confirmed by NMR and Fourier transform infrared spectroscopy (Figs. S7-S9). The hEB-DTG prodrug exhibited a UV–Vis absorbance peak at 568 nm, slightly redshifted from the 565 nm peak of free hEB (Fig. 3B). It also retained the characteristic absorbance peaks of DTG at ~254 nm and ~320 nm. Importantly, hEB conjugation dramatically improved the aqueous solubility from 0.04 mg/mL for native DTG to 9.64 mg/mL for hEB-DTG (Fig. 3C). hEB-DTG maintained strong binding ability with BSA, as demonstrated by gel retardation in Figure S10. Free BSA and hEB-DTG-bound BSA displayed a similar size distribution with an average size of ~13 nm as examined by dynamic light scattering (Fig. 3D) and transmission electron microscopy (TEM; Fig. 3E). The size of hEB-DTG bound BSA remained stable over 7 days (Fig. 3F), and only a negligible amount (0.83 %) of DTG was released over 30 days at room temperature, as detected by HPLC-MS (Figures S11 and S12), indicating an excellent stability under storage conditions. In contrast, under physiologically relevant conditions (37 °C, PBS), hEB-DTG exhibited a sustained release profile, liberating ~30 % DTG within 24 h and ~60 % over 96 h (Figs. 3G and S11). Interestingly, the release was slower in serum, probably because albumin binding protects hEB-DTG against spontaneous hydrolysis, a phenomenon consistent with other protein-binding therapeutics [38–40].

### 3.4. hEB-DTG exhibits anti-HIV activity and significantly higher concentrations in blood and LNs than DTG

**Fig. 4.**
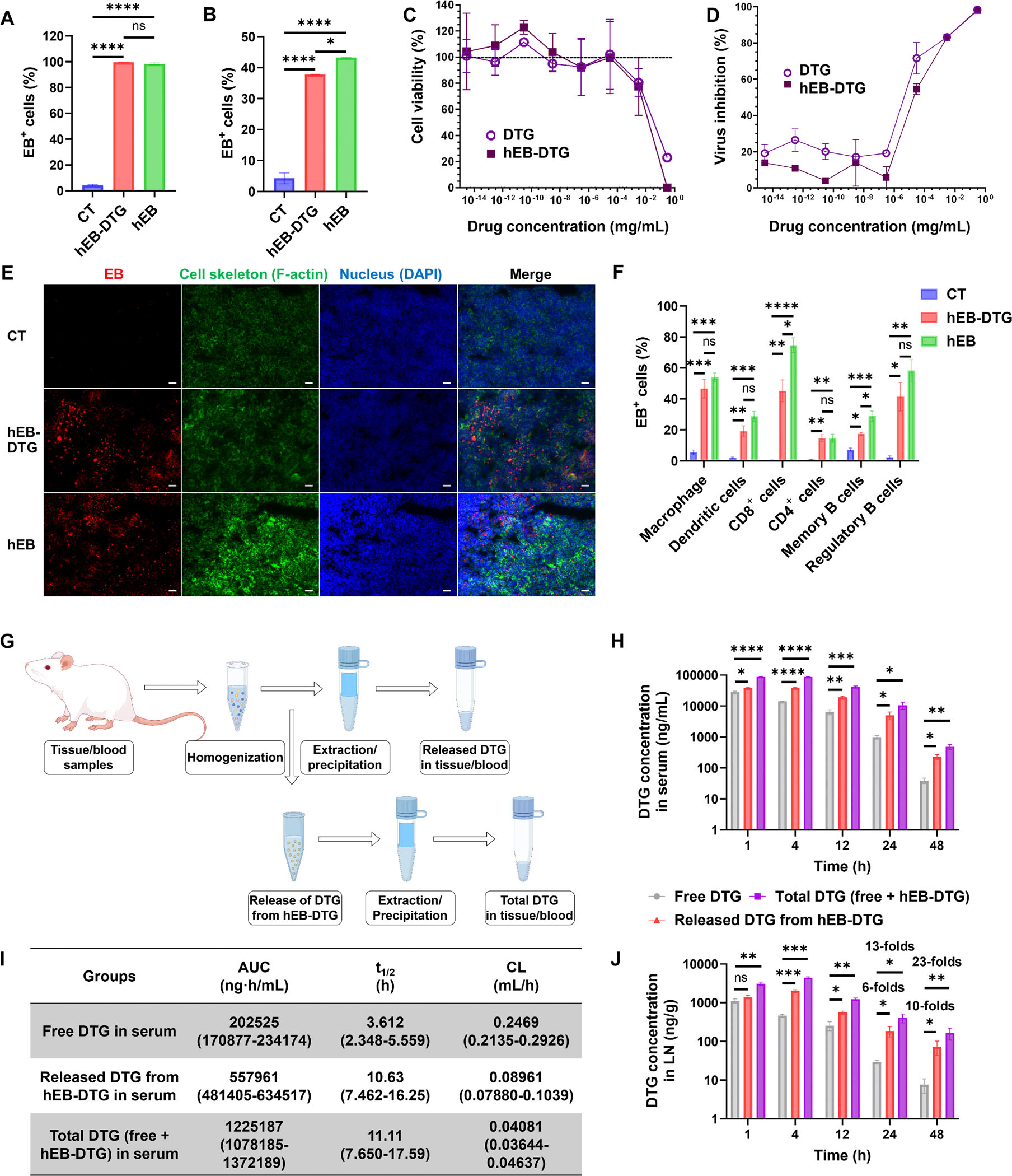
hEB-DTG showed anti-HIV activity and significantly higher concentrations in blood and LNs than DTG. (A) Cellular uptake of hEB-DTG and hEB in HEK293T cells, quantified by flow cytometry. N = 3. (B) Transport of hEB-DTG and hEB across a confluent HUVEC monolayer in a transwell assay, quantified by flow cytometry of cells in the lower chamber. N = 3. (C) Viability of TZM-bl cells after 48 h exposure to serial concentrations of free DTG or hEB-DTG. N = 3. (D) Inhibition of HIV-WT replication in TZM-bl cells treated with serial concentrations of free DTG or hEB-DTG for 48 h. N = 3. (E) Representative fluorescence microscopy images of LNs 24 h post *i.v.* injection of hEB-DTG or hEB. Scale bar: 20 μm. (F) Uptake of hEB-DTG and hEB in specific immune cell populations within LNs 8 h post *i.v.* injection, analyzed by flow cytometry. N = 3. (G) Schematic illustrating the experimental workflow and analytical methods for distinguishing released DTG from total DTG (comprising both free DTG and hEB-DTG) in blood and LN samples, drawn by Figdraw. (H&I) PK profiles of released DTG and total DTG in blood over time following a single *i.v.* injection of free DTG or hEB-DTG (H), and key PK parameters derived with a one-compartment model (I). N = 3. (J) Kinetics of released DTG and total DTG in pooled AX and IN LNs over time following a single *i.v.* injection of free DTG or hEB-DTG. N = 3. Data are presented as mean ± SEM. Statistical analysis were performed by one-way ANOVA for comparison among multiple groups, ns, no significance, *P < 0.05, **P < 0.01, ***P < 0.001, ****P < 0.0001.

Consistent with the behavior of the parent hEB molecule, the hEB-DTG prodrug was efficiently internalized by cells (Fig. 4A) and successfully transported across an endothelial cell monolayer in a transwell assay, as indicated by flow cytometry (Fig. 4B). We next evaluated the anti-HIV efficacy of hEB-DTG and compared to free DTG using TZM-bl reporter cells. TZM-bl is a HeLa-derived cell line engineered to express CD4, CCR5, and CXCR4 receptors [41], making it highly permissive to infection by most strains of HIV. The cells also contain integrated reporter genes for firefly luciferase and *E. coli* β-galactosidase under the control of an HIV-1 long terminal repeat sequence [42], which enables sensitive and accurate measurement of HIV infection. TZM-bl cells infected with HIV-WT were treated with serial concentrations of hEB-DTG or free DTG for 48 h, after which cell viability and inhibition of HIV-WT were assessed. As shown in Fig. 4C, both hEB-DTG and free DTG had minimal cytotoxicity at concentrations below 100 ng/mL. Cell viability began to decline in both groups when drug concentrations exceeded 1 μg/mL, indicating a comparable safety profile *in vitro*. In the antiviral assay, both hEB-DTG and free DTG potently inhibited HIV-WT replication at concentrations of 10 ng/mL and higher (Fig. 4D). hEB-DTG was slightly less potent than free drug, which is likely attributed to the fact that DTG must first be released from the prodrug to act, a process that is unlikely to reach completion within the 48 h assay. This controlled release profile, while resulting in less effect in a short-term *in vitro* setting, is anticipated to provide a sustained therapeutic effect *in vivo*.

Fluorescence imaging of LNs 24 h post-injection revealed that the signal intensity from hEB-DTG was comparable to that of hEB alone, confirming that the prodrug also achieves widespread distribution throughout the lymphatic system (Fig. 4E). We further characterized the cellular uptake of hEB-DTG by several major immune cell populations in the LNs using flow cytometry, including macrophages, dendritic cells (DCs), CD8^+^ T cells, CD4^+^ T cells, memory B cells, and regulatory B cells (Fig. S3). As shown in Fig. 4F, significant EB signals were detected in all cell types analyzed, with levels slightly lower than those in the hEB-only group. Notably, macrophages exhibited the strongest fluorescence intensity among all cell types.

We next conducted a pharmacokinetic (PK) study to evaluate whether hEB-DTG enhances DTG retention in the blood and improves lymphatic distribution compared to free DTG (Fig. 4G). Given the expected low DTG concentrations in LNs following free DTG administration, AX and IN LNs were pooled for analysis. An HPLC-MS method was established for quantitative measurement of DTG (Figures S11–S13). Samples from the prodrug group contained both released free DTG and intact hEB-DTG (Figs. S11–S13). Our pilot experiments revealed that albumin-bound hEB-DTG was resistant to hydrolysis under basic conditions, hindering complete release of DTG from the prodrug (Fig. 3G). This issue was effectively resolved by pretreatment with guanidine hydrochloride at 80 °C, which denatures albumin, releases the bound prodrug, and enables complete hydrolysis of hEB-DTG to free DTG (Figs. S11–S13).

Following a single *i.v.* injection, hEB-DTG exhibited a markedly improved PK profile (Fig. 4H). At 1 h post-injection, the concentration of released DTG in the hEB-DTG group was slightly higher than that of the free DTG group, while the total DTG concentration was 2.1-fold greater. Thereafter, DTG levels in the free DTG group declined rapidly, whereas those in the hEB-DTG group were sustained over time. From 4 h onward, the plasma concentrations of released DTG were 1.8- to 4.9-fold higher, and total DTG concentrations were 5.2- to 11.4-fold higher in the hEB-DTG group compared to free DTG. The persistent difference between total and released DTG concentrations suggests a slow and continuous release of DTG from the prodrug. PK analysis using a one-compartment model (Fig. 4I) further supported these findings. The hEB-DTG group demonstrated a significantly greater area under the curve (AUC), an extended half-life (t_1/2_), and reduced clearance (CL) for both released and total DTG, confirming enhanced systemic exposure and prolonged circulation.

The distribution of DTG to LNs was markedly enhanced by the hEB-DTG prodrug (Fig. 4J). In mice treated with free DTG, LN concentrations were only 1/5 to 1/30 of the corresponding blood levels at all time points, confirming poor lymphatic distribution. Moreover, LN concentrations declined rapidly in parallel with the decreases in blood levels. In sharp contrast, mice treated with hEB-DTG exhibited peak LN concentrations at 4 h post-injection, followed by a gradual decline. The concentration of released DTG in LNs was approximately 5-fold higher than that of free DTG group at 24 h and nearly 10-fold higher at 48 h. When total DTG was considered, the enhancement was even more pronounced, with ~12-fold and ~22-fold higher LN levels at 24 and 48 h, respectively. Collectively, these results demonstrate that hEB-DTG profoundly alters the PK profile of DTG, enabling sustained and high-level drug exposure within the lymphatic system. This provides a strong PK rationale for evaluating the anti-HIV efficacy of hEB-DTG in the lymphoid tissues of HIV-infected humanized mouse models in future studies.

## 4. Conclusions

In this study, we demonstrate that EB, through its strong albumin-binding affinity, promotes albumin-mediated cellular uptake and trans-endothelial transcytosis, thereby enabling efficient and widespread delivery to lymphatic tissues. The hEB prodrugs markedly increased drug concentrations and retention in key organs, achieving up to a 10-fold enhancement in LNs and GALT. Importantly, *i.v.* administration of hEB-based prodrugs ensured uniform drug distribution throughout the lymphatic system—a clear advantage over *s.c.* injection, which primarily targets local draining LNs.

Furthermore, we developed and evaluated an hEB-conjugated ARV prodrug, hEB-DTG, which retained potent anti-HIV activity *in vitro* with a favorable safety profile. Notably, *in vivo* PK studies revealed that hEB-DTG achieved approximately 5-fold higher systemic total DTG exposure and profoundly enhanced lymphatic delivery, with total DTG concentrations in LNs exceeding 20-fold those obtained with free DTG at 48 h post-injection. hEB-DTG was efficiently internalized by all major immune cell subsets within LNs, including macrophages, DCs, CD8^+^ T cells, CD4^+^ T cells, memory B cells, and regulatory B cells. Collectively, these findings underscore the translational potential of our therapeutic strategy: hEB-conjugated ARV prodrugs substantially increase drug concentrations within the lymphatic system—the principal HIV reservoir—offering a promising avenue toward HIV eradication.

## Supplementary Material

1

Supplementary material associated with this article can be found, in the online version, at doi:10.1016/j.actbio.2025.11.017.

## Statement of significance:

A major barrier to curing HIV infection is the persistence of viral reservoirs in lymphatic tissues, which current ARV therapies fail to eliminate due to poor drug penetration. To overcome this limitation, we developed a prodrug strategy that harnesses the natural trafficking of albumin to the lymphatic system. In this approach, ARVs are conjugated to EB, a high-affinity albumin-binding dye, to generate EB–ARV prodrugs that hijack albumin’s endogenous transport pathways. After intravenous administration, these conjugates rapidly associate with circulating albumin, which mediates their transcytosis across endothelial barriers and directs their accumulation in lymph nodes and other lymphoid tissues. This strategy substantially enhances ARV exposure at the primary sites of viral persistence, representing a promising and potentially transformative approach for eradicating HIV reservoirs and advancing toward a functional cure.

## List of Abbreviations

AAALAC: Association for Assessment and Accreditation of Laboratory Animal Care
ART: antiretroviral therapy
ARV: antiretroviral
AUC: area under curve
AX LN: axillary lymph node
BSA: bovine serum albumin
cART: combination antiretroviral therapy
CL: clearance
CE LN: cervical lymph node
DC: dendritic cell
DMEM: Dulbecco’s modified Eagle’s medium
DTG: dolutegravir
EB: Evans Blue
EEA1: early endosome antigen 1
FBS: fetal bovine serum
GALT: gut-associated lymphoid tissue
hEB: half-truncated Evans Blue
hEB-DTG: half-truncated Evans Blue conjugated dolutegravir prodrug
hEB: platin half-truncated Evans Blue conjugated cisplatin prodrug
HIV: human immunodeficiency virus
*i.v*.: intravenous
ICP-MS: inductively coupled plasma-mass spectrometer
IN LN: inguinal lymph node lipoplatin cisplatin-loaded liposomes
LN: lymph node
LPS: lipopolysaccharide
MD LN: mediastinal lymph node
MFI: mean fluorescence intensity
MS LN: mesenteric lymph node
NMR: nuclear magnetic resonance
NNRTI: non-nucleoside reverse-transcriptase inhibitor
NP: nanoparticle
NRTI: nucleoside reverse-transcriptase inhibitor
one-way ANOVA: one-way analysis of variance
PK: pharmacokinetic
*s.c.*: subcutaneous
t_1/2_: half-life
