# Supplementary material for: Harnessing albumin as a carrier for the delivery of anti-HIV drugs to the lymphatic system
Source: Acta Biomater. Author manuscript; Available in PMC 2026 Jul 7. (PMC13338763; doi:10.1016/j.actbio.2025.11.017)
Supplement: 1 [file NIHMS2186321-supplement-1.docx]

**Harnessing Albumin as a Carrier for the Delivery of Anti-HIV Drugs to the Lymphatic System**

*Ziqian Zhang^1,2^, Yixian Huang^1,2^, Raymond E. West III^3^, Deepika Mahesh^4^, Patrick Joseph Oberly^3^, Shichen Li^1,2^, Zhangyi Luo^1,2^, Yuang Chen^1,2^, Haozhe Huang^1,2^, Daniel J. Bain^5^, Thomas D Nolin^3^, Moses T. Bility^6^,*

*Robbie B Mailliard^4^, and Song Li^1,2 *^*

^1^Center for Pharmacogenetics, Department of Pharmaceutical Science, University of Pittsburgh School of Pharmacy, Pittsburgh, PA, USA

^2^UPMC Hillman Cancer Center, University of Pittsburgh, Pittsburgh, PA, USA

^3^Small Molecule Biomarker Core, University of Pittsburgh School of Pharmacy, Pittsburgh, PA, USA

^4^Department of Medicine, University of Pittsburgh School of Medicine, Pittsburgh, PA, USA

^5^Department of Geology and Environmental Science, University of Pittsburgh, Pittsburgh, PA, USA

^6^Department of Microbiology, Howard University College of Medicine, Washington, DC, USA

**Author for correspondence: Song Li, M.D., Ph.D.*

*Email: sol4@pitt.edu*

*Telephone: +1 412-383-7976*

*Fax: +1 412-648-1664*

*Postal address: 313 Salk Pavilion, 355 Sutherland Drive, Pittsburgh, PA, USA*

# **Supplementary Figures**


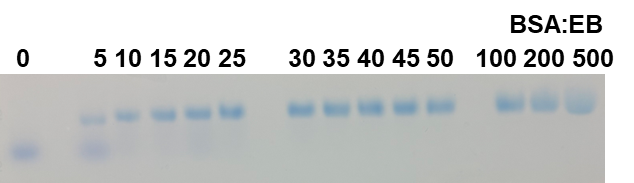


Figure S1. Agrose gel retardation study of EB mixed with different amount of BSA (weight ratio).


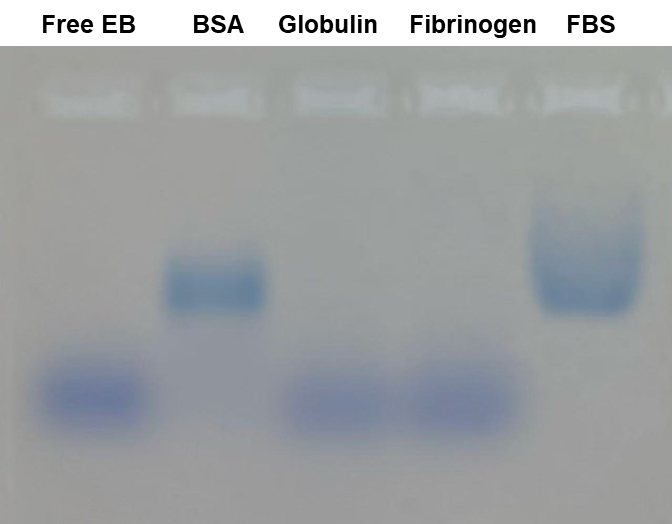


Figure S2. Agrose gel retardation study of EB mixed with different serum proteins.


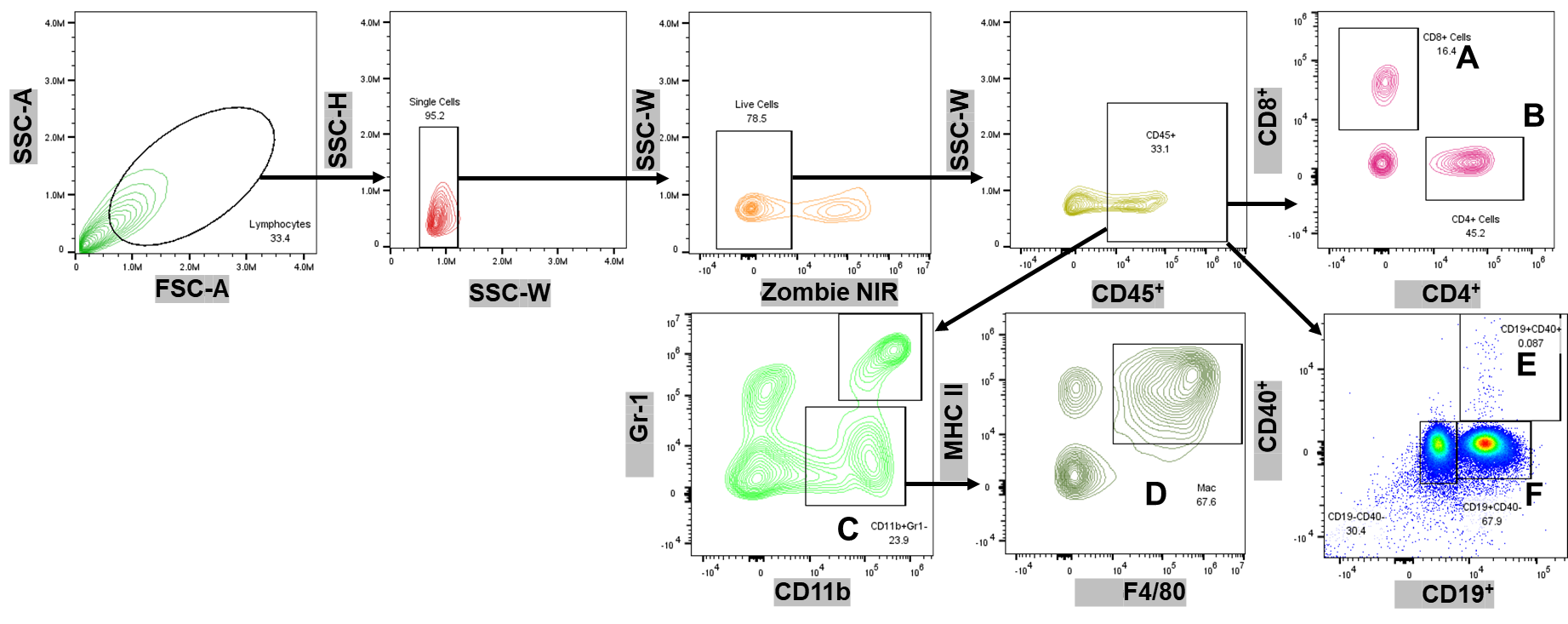


Figure S3. Gating strategy for different types of cells in LN.

Gating strategies for CD8^+^ T cells (A), CD4^+^ T cells (B), dendritic cells (C), macrophages (D), memory B cells (E) and regulatory B cells (F) in LN.

**hEB-platin**

**PROTON D_2_O**

Figure S4. ^1^H-NMR spectrum of hEB-platin.


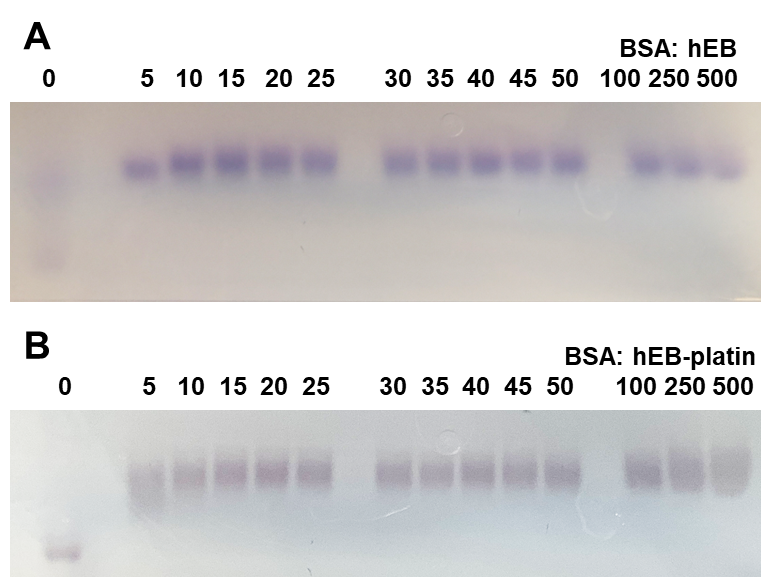


Figure S5. Agrose gel retardation study of hEB (A) or hEB-platin (B) mixed with different amount of BSA (weight ratio).

Figure S6. Standard curve for measurement of Pt content by ICP-MS.

Figure S7. ^1^H-NMR spectrum of Cl-DTG.

Figure S8. ^1^H-NMR spectrum of hEB-DTG.


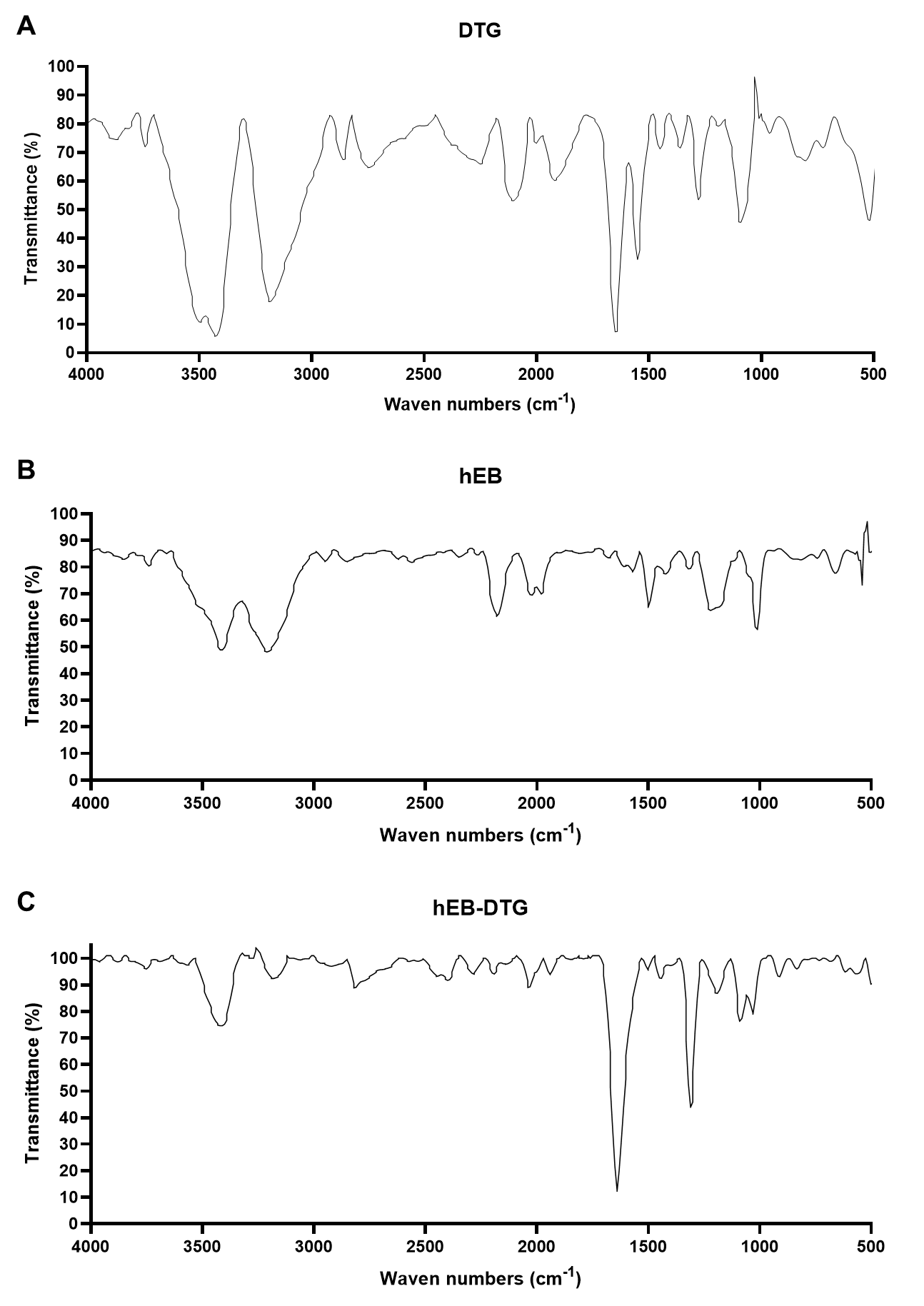


Figure S9. Fourier transform infrared spectroscopy spectrum of DTG (A),

hEB (B) and hEB-DTG (C).


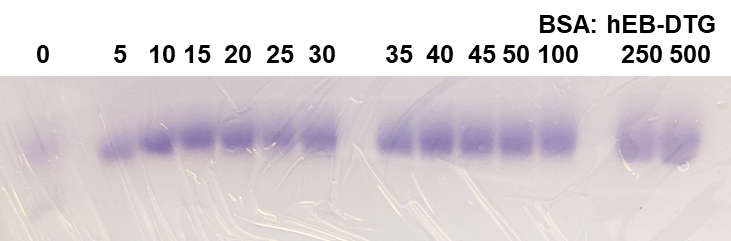


Figure S10. Agrose gel retardation study of hEB-DTG mixed with different amount of BSA (weight ratio).


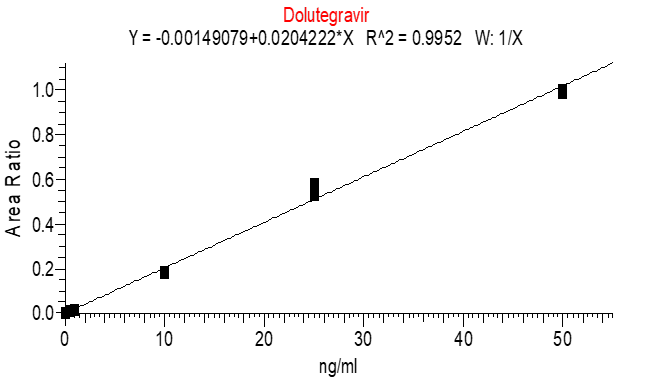


**Area Ratio**

**ng/mL**

Figure S11. Standard curve for measurement of DTG concentration by HPLC-MS.

Figure S12. Percentage of DTG released from hEB-DTG under storage condition (RT, room temperature, aq.) and heated condition (80 ℃, aq.).

Figure S13. Recovery of DTG and hEB-DTG at 80 ℃ homogenated lymph node tissue suspension with guanidine hydrochloride.
